# Smoking Significantly Impacts Persistence Rates in Embolized Pulmonary Arteriovenous Malformations in Patients with Hereditary Hemorrhagic Telangiectasia

**DOI:** 10.1148/radiol.2019180978

**Published:** 2019-07-30

**Authors:** Mustafa M. Haddad, Emily C. Bendel, William S. Harmsen, Vivek N. Iyer, Sanjay Misra

**Affiliations:** From the Department of Radiology (M.M.H., E.C.B., S.M.), Department of Biomedical Statistics and Informatics (W.S.H.), and Department of Pulmonary and Critical Care Medicine (V.N.I.), Mayo Clinic, 200 First St SW, Rochester, MN 55905.

## Abstract

**Background:**

Embolization is the standard of care for treatment of pulmonary arteriovenous malformations (PAVMs). Persistence of PAVMs after embolization occurs for undefined reasons but may include inflammation related to smoking in dysregulated angiogenesis.

**Purpose:**

To determine whether patients with hereditary hemorrhagic telangiectasia (HHT) who smoke tobacco are more prone to PAVM persistence after embolization.

**Materials and Methods:**

Patients with HHT treated for PAVMs between January 2000 and August 2017 were retrospectively identified. Only PAVMs with no previous treatment and patients with both clinical and imaging follow-up were included. Age, sex, PAVM characteristics (size, complexity, and location), embolization material used, microcatheter type, smoking history, active tobacco use, and other risk factors for arterial disease were analyzed by using a multivariate Cox proportional hazards model to determine risk factors for persistence.

**Results:**

Five-year persistence-free survival rates in nonsmokers, smokers of 1–20 pack-years, and smokers of more than 20 pack-years were 12.2%, 21.9%, and 37.4% respectively. Smokers with more than 20 pack-years relative to nonsmokers had greater risk of persistence after adjusting for arterial feeder size (hazard ratio, 3.8; 95% confidence interval [CI]: 1.5, 10.0; *P* = .007). Patients who reported active tobacco use at the time of PAVM embolization had a 5-year cumulative incidence of persistence of 26.3% compared with 13.5% in inactive smokers. After adjusting for arterial feeder size, the risk of persistence was greater in tobacco users versus inactive smokers at the time of treatment (hazard ratio, 2.4; 95% CI: 1.2, 4.7; *P* = .01).

**Conclusion:**

Smoking is associated with pulmonary arteriovenous malformation persistence after embolization in patients with hereditary hemorrhagic telangiectasia.

*Online supplemental material is available for this article.*

See also the editorial by Trerotola and Pyeritz in this issue.

## Graphical abstract

**Figure:**
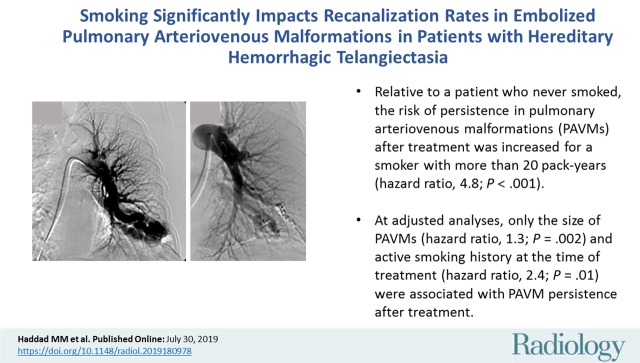

**Summary**

Patients with hereditary hemorrhagic telangiectasia and active tobacco use have higher rates of pulmonary arteriovenous malformation persistence after percutaneous embolization.

**Key Points**

■ Relative to a patient who never smoked, the risk of persistence in pulmonary arteriovenous malformations (PAVMs) after treatment was increased for a smoker with more than 20 pack-years (hazard ratio, 4.8; *P* < .001).
■ At adjusted analyses, only the size of PAVMs (hazard ratio, 1.3; *P* = .002) and active smoking history at the time of treatment (hazard ratio, 2.4; *P* = .01) were associated with PAVM persistence after treatment.

## Introduction

Hereditary hemorrhagic telangiectasia (HHT) is an autosomal dominant vascular disease characterized by the presence of multiorgan arteriovenous malformations (AVMs) including pulmonary AVMs (PAVMs) (1). PAVMs can occur in 30%–50% of patients with HHT (2). PAVMs can cause numerous systemic complications mainly related to shunting (hypoxemia) and paradoxical embolism (brain and other visceral organ abscesses, and ischemic stroke) (3). The treatment of PAVMs has evolved over the past few decades from surgical resection to endovascular embolization, which is currently the standard of care for PAVMs (4). Endovascular embolization is performed with a variety of coils and plug-like devices. Re-establishment of a vascular connection between an arterial feeder and draining vein after embolization or persistence occurs in up to 15%–25% of all patients after embolization and is one of the most common problems after endovascular embolization (5–8).

Many procedural and anatomic factors have been described as risk factors for persistence, including PAVM size, PAVM complexity (multiplicity of feeding arteries and draining veins), lack of properly packed coils, inadequate distal coil embolization, and development of new arterial feeders (7,9). An understanding of these risk factors has led to specific changes in procedural technique and equipment design to minimize persistence (1). For instance, plug occlusion devices tend to have decreased persistence rates compared with traditional coil embolization (10). Larger and more complex PAVMs are also treated differently compared with simple PAVMs because they require careful identification and embolization of each arterial feeder branch (5). However, a number of persistence events continue to occur for undefined reasons. This may be because inflammation, which has a role in dysregulated angiogenesis, and elevated inflammatory markers are noted in patients with AVMs and patients with HHT (11). Smoking is one of the most critical environmental factors contributing to inflammation because smoking disrupts vascular angiogenesis and remodeling (12). Inhaling tobacco smoke causes the release of more than 3000 chemicals into the respiratory system, which can cause vascular damage and remodeling (13). The purpose of our study was to determine whether smoking increases the risk of PAVM persistence after percutaneous embolization in patients with HHT.

## Materials and Methods

**Figure 1:**
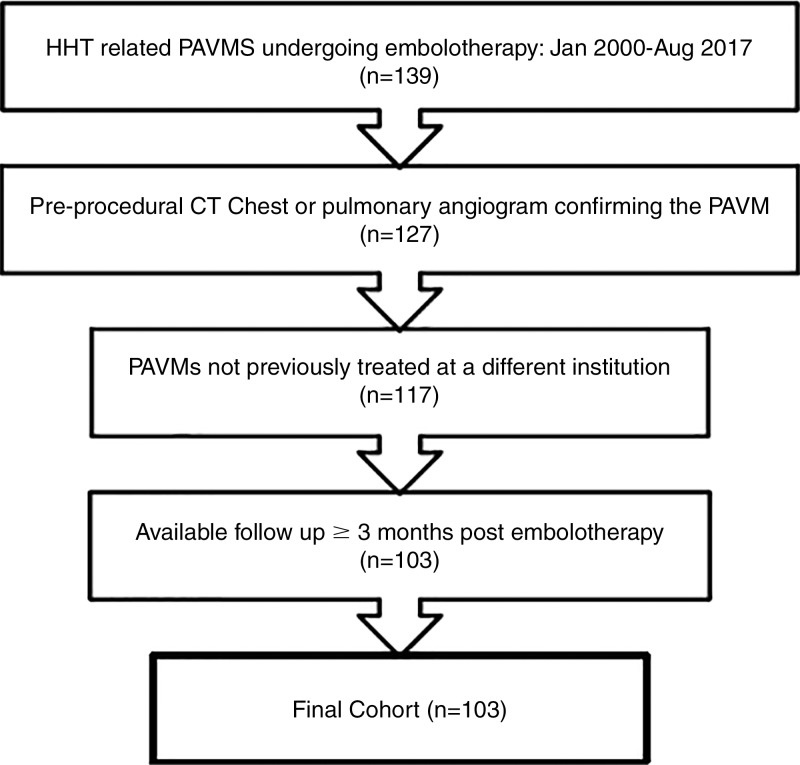
Inclusion flowchart. HHT = hereditary hemorrhagic telangiectasia, PAVM = pulmonary arteriovenous malformation.

This retrospective study was approved by our institutional review board. This was deemed as a minimal risk study by the institutional review board and informed consent was waived. All patients with HHT who were treated for pulmonary AVMs at our institution between January 2000 and August 2017 were identified. We only included patients who definitely had HHT as defined by the presence of three or more Curaçao criteria (14). PAVMs were identified at either contrast agent–enhanced chest CT or pulmonary arteriography. A PAVM was defined by the presence of a dilated arterial vessel connected to a dilated draining vein by a PAVM sac (15). All PAVM embolizations were performed by staff members of the Division of Vascular and Interventional Radiology (E.C.B. and S.M., with an average of 22 years of experience in PAVM embolizations; range, 1–33 years). Adequate imaging follow-up required a contrast-enhanced chest CT or pulmonary arteriography performed at least 3 months after embolization and yearly after the embolization. Patients with PAVMs who were previously treated at an outside institution and patients without adequate clinical and imaging follow-up were excluded, leaving a final cohort of 103 patients (Fig 1). Demographic, clinical, procedural, anatomic, and treatment outcomes were obtained by using our institution’s electronic medical record database.

### PAVM Embolization Procedural Details

Access was typically gained via the right common femoral vein. Prophylactic antibiotics were not used. Use of local or general anesthesia depended on patient complexity and operator preference. After placement of a 5–8-F introducer sheath, an angiographic catheter (APC; Cook Medical, Bloomington, Ind) was used to access the pulmonary trunk. A diagnostic pulmonary arteriogram was then obtained in the involved side on the basis of the location of the PAVMs by using a 5–7-F pigtail catheter placed in the main pulmonary artery with a contrast agent injection of 20–25 mL/sec for a total volume of 40–50 mL. Simple PAVMs were classified as a single artery feeding the malformation, whereas complex PAVMs were classified as multiple segmental arteries feeding the malformation (16).

**Figure 2:**
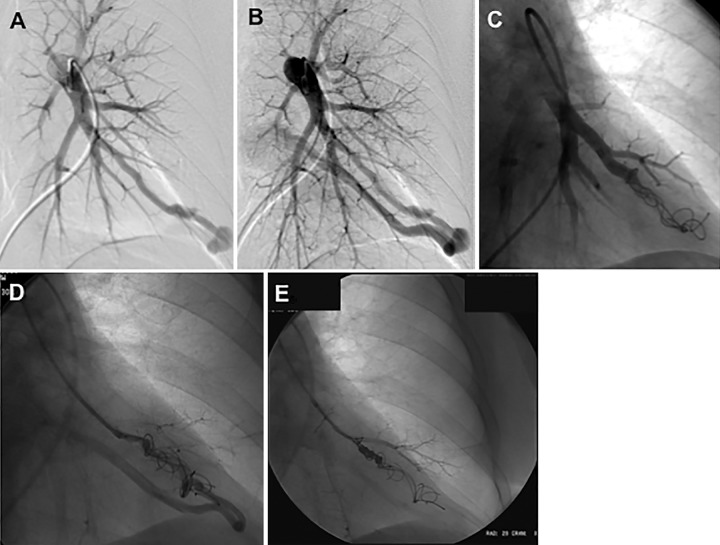
Images in a 52-year-old woman with hereditary hemorrhagic telangiectasia demonstrate on left pulmonary artery angiogram an arteriovenous malformation (AVM) at, *A*, *B*, the left base. *C*, Selective angiogram after embolization of the AVM by using coils. *D*, *E*, Angiograms show embolization for persistence treated with repeat embolization performed 2 years after initial embolization.

Embolization of the arterial feeder was then performed with either coils or plugs on the basis of operator preference. Coils or plugs were placed starting from the distal-most aspect of the arterial feeder in as close proximity to the PAVM sac as possible (Fig 2). Embolization agents included Nester and Tornado coils (Cook Medical), Amplatzer plugs (St Jude Medical, St Paul, Minn), Ruby coils (Penumbra, Alameda, Calif), Hilal-Silver coils (Cook Medical), fiber coils (Boston Scientific, Marlborough, Mass), helical coils (Medtronic, Minneapolis, Minn), and microvascular plugs (MVP; Medtronic). Use of a microcatheter and choice of embolization material was determined by the operator at time of procedure. After embolization, selective digital subtraction arteriography was performed to ensure adequate embolization of the PAVM. The amount of contrast agent used depended on the diameter of the inflow artery and flow within the AVM. If continued perfusion was demonstrated at postembolization arteriography, further coils or plugs were placed until the final digital subtraction arteriogram showed complete embolization typically performed by using the same rate and volume of contrast agent as the diagnostic angiography.

### Primary and Secondary Persistence of PAVMs after Embolization

**Figure 3:**
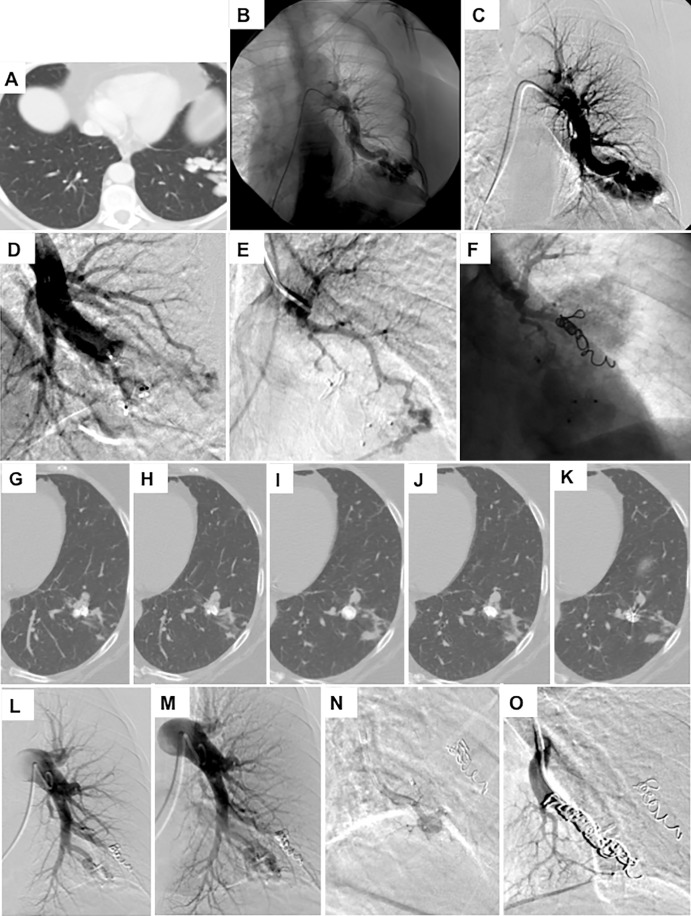
Images in a 68-year-old woman with hereditary hemorrhagic telangiectasia (HHT) and a smoking history of 40 pack-years demonstrate, *A*, a complex arteriovenous malformation (AVM) at the left base on intravenous contrast-enhanced chest CT scan. *B*, *C*, Left pulmonary artery angiograms show the AVM. *D*, Image after embolization and after placement of an Amplatzer plug. *E*, A selective angiogram of the second arterial blood supply to the AVM. *F*, Angiogram after coil embolization. *G*–*K*, Selective 1.5-mm intravenous contrast-enhanced axial CT images show persistence of the AVM 1 year later. *L, M*, Left pulmonary artery angiograms show persistence. *N*, Selective catherization of the artery supplying the AVM. *O*, Angiogram after coil embolization of the AVM.

Re-establishment of an arteriovenous connection in a PAVM after embolization is known as persistence. If this occurs after the first embolization procedure it is termed primary persistence whereas if it occurs after a second or further re-embolization procedure it is termed secondary persistence. Persistence can occur by several mechanisms including blood flow through the embolization coil or plug (ie, recanalization), development of a new pulmonary arteriovenous connection, incomplete primary treatment because of untreated feeders, development of a systemic artery-pulmonary shunt (eg, bronchial artery to pulmonary shunting), and combination of all factors (7). Follow-up imaging to determine persistence consisted of CT angiography of the chest (Fig 3, Appendix E1 [online]). Pulmonary arteriography was performed only when patients were referred for repeat embolization. The decision to re-embolize lesions that showed primary persistence was on the basis of clinical and radiologic criteria such as size of the intrapulmonary shunt, return of hypoxia, and patient or physician preference. Data regarding each persistence event and additional data including subsequent embolizations and the occurrence of any associated complications were recorded. Complications were defined by using the Society of Interventional Radiology standards (17).

### Analysis of Smoking Status

Smoking status was analyzed both as a dichotomous variable (never smoker vs any history of smoking) and also as an ordinal dose-response relationship (never smoker vs ≤20 pack-years vs >20 pack-years history of smoking). We also grouped patients on the basis of whether or not they reported active smoking at the time of the procedure.

### Statistical Analysis

Data for discrete variables are reported as number with percentage and for continuous variables as median with range. Time to persistence was calculated from date of embolization to the date of the chest CT or pulmonary arteriography that showed persistence, death, or last contact. Estimates of survival free of persistence were calculated by using the Kaplan-Meier method. A Cox proportional hazards regression model was used to assess variable associations with the risk of persistence, with the results reported as hazard ratios and 95% confidence intervals (CIs). Models accounted for multiple-treated sites in a given patient to decrease the weighting of patients with multiple PAVMs that were treated. The weight assigned for a patient was the inverse of log of the number of PAVMs for the patient. A multiple variables model was examined by using backward selection, including as candidate variables all with a univariate *P* value of less than .20. *P* values less than .05 were considered to indicate statistical significance. All analyses were performed by using statistical software (SAS version 9.4; SAS Institute, Cary, NC).

## Results

From January 2000 to August 2017, a total of 103 consecutive patients with HHT (37.9% men [39 of 103]; median patient age, 52 years [age range, 11–90 years]) underwent embolization in 373 PAVMs in 151 distinct procedures. Median follow-up was 6.2 years (range, 0.02–14.9 years) from the date of each respective primary PAVM embolization, and median PAVM arterial feeder size was 3 mm (range, 1–13 mm). Demographic data are shown in Table 1. Characteristics associated with the individual PAVM (*n* = 373) included location, tobacco use, use of embolization agent, and use of microcatheter (Table 2). Technical success, defined as complete occlusion of the inflow artery supplying the AVM demonstrated by using a hand injection of contrast agent, was achieved in all but two procedures (two of 151; 1.3%). In these two procedures, complications occurred that prevented the completion of the embolization procedure. Complex PAVMs were treated in 10.4% of PAVMs (39 of 373; Table 2) with no difference in rates of complex PAVMs between the group with more than 20 pack-years versus the nonsmoking group or group with 20 pack-years or less (*P* = .43; Table 1).

**Table 1:**
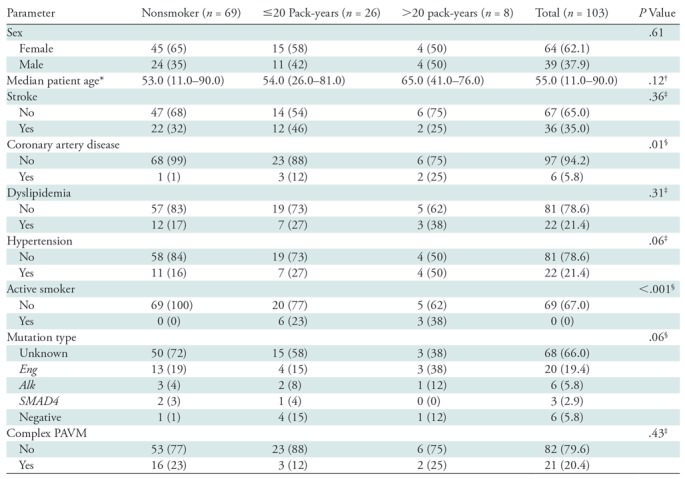
Characteristics of Patients with Hereditary Hemorrhagic Telangiectasia and Pulmonary Arteriovenous Malformations

Note.—There were 103 patients with hereditary hemorrhagic telangiectasia. Data in parentheses are percentages unless otherwise indicated. PAVM = pulmonary arteriovenous malformation.

* Data in parentheses are range.

^†^ Kruskal-Wallis test.

^‡^ χ^2^ test.

^§^ Fisher exact test.

**Table 2:**
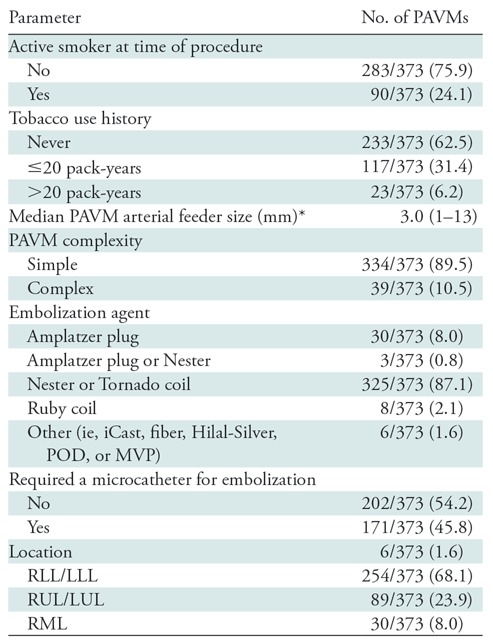
Characteristics of Pulmonary Arteriovenous Malformations in Patients with Hereditary Hemorrhagic Telangiectasia

Note.—Unless otherwise indicated, data in parentheses are percentages. There were 373 pulmonary arteriovenous malformations in 103 patients with hereditary hemorrhagic telangiectasia. LLL = left lower lobe, LUL = left upper lobe, MVP = Microvascular Plug (Medtronic), PAVM = pulmonary arteriovenous malformation, POD = Penumbra Occlusion Device (Penumbra, Alameda, Calif), RLL = right lower lobe, RML = right middle lobe, RUL = right upper lobe.

* Data in parentheses are range.

### Primary and Secondary Persistence

Primary persistence occurred in 61 PAVMs, and the 5-year cumulative incidence of persistence was 17.3% (95% CI: 12.8%, 21.6%). Recanalization was the most frequent cause of persistence, occurring in 64% (39 of 61) of all persistence events (Table 3). There were five recanalization events in the group with more than 20 pack-years, 16 recanalization events in the group with 20 pack-years or less and 18 recanalization events in the nonsmoking group. The 5-year cumulative incidence of persistence for nonsmokers, smokers with 20 pack-years or less, and smokers with greater than 20 pack-years was 12.2% (95% CI: 7.1%, 17.1%), 21.9% (95% CI: 13.3%, 30.6%), and 37.8% (95% CI: 13.0%, 58.5%), respectively. Overall, there was an association of smoking history with risk of persistence (*P* < .001) relative to a nonsmoker. The risk was increased for a smoker with more than 20 pack-years (hazard ratio, 4.8; 95% CI: 2.2, 10.4; *P* < .001) and the risk was nonsignificantly increased for a smoker with 20 pack-years or less (hazard ratio, 1.2; 95% CI: 0.6, 2.6; *P* = .63). A second embolization procedure was performed in 64% of persistence cases (39 of 61). Among these 39 PAVMs that were re-embolized, repeat persistence or secondary persistence occurred in 18% (seven of 39). The 1-year cumulative incidence of secondary persistence was 5.9%.

**Table 3:**
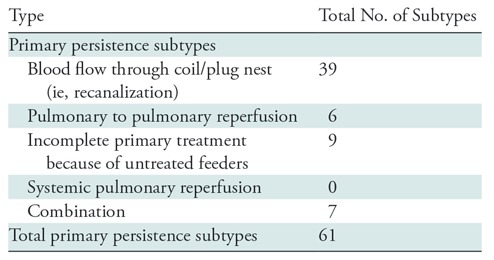
Primary Persistence Subtypes

By examining smoking status at the time of the embolization, the 5-year cumulative probability of persistence in patients who were active smokers was 26.3% (95% CI: 16.2%, 36.6%) compared with 13.5% (95% CI: 8.7%, 18.0%) in inactive smokers.

### Risk Factors Associated with PAVM Persistence

In a multiple variable model that included arterial feeder size in addition to smoking history there was an overall association of cumulative smoking history with the risk of persistence (*P* = .02; Table 4). Compared with a nonsmoker; a patient with a history of more than 20 pack-years had a nearly fourfold increased risk (hazard ratio, 3.8; 95% CI: 1.5, 10.0; *P* = .007). By using a model that included arterial feeder size and smoking status at embolization, an active smoker at embolization was at an increased risk of persistence (hazard ratio, 2.4; 95% CI: 1.2, 4.7; *P* = .01; Table 4).

**Table 4:**
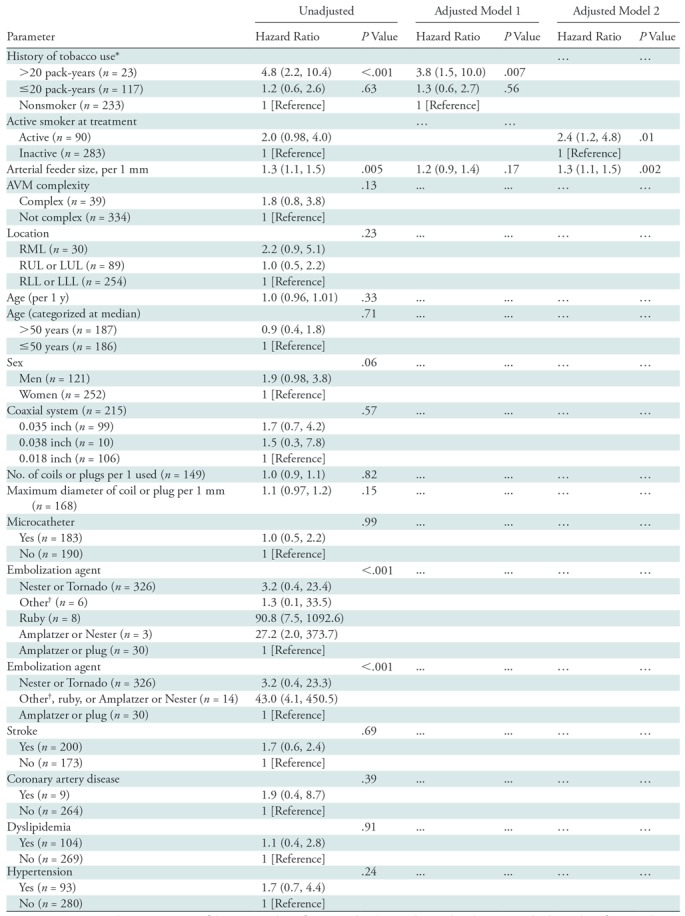
Univariable and Multivariable Regression Models for Persistence of Pulmonary Arteriovenous Malformation Following Treatment

Note.—Data in parentheses are 95% confidence intervals. Reference, in brackets, indicates that the associated value is the reference value. AVM = arteriovenous malformation, LLL = left lower lobe, LUL = left upper lobe, RLL = right lower lobe, RML = right middle lobe, RUL = right upper lobe.

* The overall association of smoking history with the risk of persistence was univariable model, *P* value less than .001, and multivariable model, *P* value equal to .02.

^†^ Includes iCast (Atrium Getinge, Wayne, NJ), fiber, Hilal-Silver (Cook Medical), and MVP (Medtronic) coils.

### Repeat Embolization and Secondary Persistence in Smokers

A repeat embolization procedure was performed in 83% (10 of 12) of smokers with a history greater than 20 pack-years in nine distinct procedures versus 44% (10 of 23) in the patients in the group with 20 pack-years or less in six distinct procedures versus 73% (19 of 26) in the nonsmoking group who underwent repeat embolization in 13 distinct procedures.

Secondary persistence occurred in 18% (seven of 39) of procedures. Among the 10 PAVMs in the group with more than 20 pack-years, five patients (50%) demonstrated secondary persistence compared with one patient each in the group with 20 pack-years or less (10%) and the nonsmoking group (5%).

### Procedural Complications

Three complications were noted in the 151 procedures (2.0%). The most severe complication (Society of Interventional Radiology grade D) consisted of an embolization that resulted in an infected pulmonary cavity, which was resolved after treatment with antibiotics. This infected cavity required surgical intervention for treatment. The second major complication (Society of Interventional Radiology grade C) consisted of nondetachable coil migrating to the right middle cerebral artery. These coils were successfully retrieved with the assistance of neurointerventional specialists. The patient had left upper extremity weakness and ataxia, which resolved after 2 months of physical therapy. Finally, the minor complication (Society of Interventional Radiology grade B) consisted of prolonged hypotension during a procedure that caused premature termination of the procedure. The cause of hypotension was not identified, and the procedure was completed on the following day with no complications. There were no procedure-related deaths.

## Discussion

Re-establishment of a vascular connection between an arterial feeder and draining vein after embolization of pulmonary arteriovenous malformations (PAVMs), defined as PAVM persistence, occurs in up to 15%–25% of patients and is one of the most common problems after endovascular embolization (5,6). In our study, we evaluated the role of smoking as a potential important contributor to persistence of PAVM after embolization. Our study shows that patients with hereditary hemorrhagic telangiectasia (HHT) with a smoking history greater than 20 pack-years have higher rates of primary persistence after PAVM embolization compared with nonsmokers (37.8% vs 12.2%, respectively). The overall 5-year cumulative primary persistence rate was 17.3%. The subgroup of patients with more than 20 pack-years of smoking had the highest risk of persistence (52%; 12 of 23). Similarly, the 5-year cumulative probability of primary persistence was 38% in this group compared with 22% and 12% for with less than 20 pack-years of smoking history and nonsmoking patient groups, respectively.

Rates of secondary persistence were also higher in the group with greater than 20 pack-years. The overall 5-year cumulative persistence rate in this study was 17.3%, which does not differ from the literature, which ranges around 15%–25% (5–8). However, the rate of secondary persistence was 50% in this group compared with 10% and 5% for the group with 20 pack-years or less and the nonsmoking group, respectively. The 5-year cumulative probability of secondary persistence could not be calculated because of lack of adequate follow-up after the repeat embolization procedure. Finally, patients who reported active tobacco use at the time of the procedure were more likely to have PAVM persistence compared with patients who did not report using tobacco at the time of procedure. These observations point to a dose-response and temporal relationship between smoking and coronary artery disease and risk of PAVM persistence in patients with HHT after embolization.

There are several potential hypotheses that could explain our observations. There is evidence to link smoking to neoangiogenesis, accelerated atherosclerosis, and endothelial injury. Chemicals in tobacco smoke have wide ranging effects on the vascular endothelium that include the creation of a prothrombotic, proinflammatory milieu with increased oxidative stress that predisposes the patient to vessel wall injury and accelerated atherosclerosis (18). A reduced number of circulating endothelial progenitor cells (involved in vessel wall repair) has also been reported in patients who smoke (19). Nicotine itself has been shown to have proangiogenic effects on the vascular endothelium by promoting endothelial cell proliferation, migration, and capillary tube formation through its interactions with the endothelial nicotinic acetylcholine receptors (20). Smoking has been found to increase serum and placental levels of vascular endothelial growth factor-A, known as VEGF-A, which is the primary driver of angiogenesis (21). It is well established that patients with HHT have high serum levels of VEGF-A (22). Thus, there are several plausible explanations for why smoking could drive vessel wall injury, neoangiogenesis, and postembolization PAVM persistence in patients with HHT.

Additionally, PAVMs themselves appear to have elevated baseline inflammatory markers, which is evidenced by increased influx of macrophages (23). Smoking-induced endothelial injury triggering abnormal vascular remodeling may further exacerbate this elevated baseline inflammatory profile. An additional pathway may involve local and systemic hypoxia caused by smoking-mediated lung injury, which may result in neoangiogenesis through the hypoxia-inducible factor-1 α pathway (24). Therefore, there are plausible explanations for the findings of temporal and dose-response correlations between smoking and persistence (8).

This study did not demonstrate a difference in the rates of persistence between the two main embolization agents currently used, Nester and Tornado coils (Cook Medical) and Amplatzer plugs (St Jude Medical) (25). Tau et al (10) showed that rates of persistence were lower with plugs than with coils. Studies (26,27) have shown improved efficacy with plugs and have also shown that the combination of coils and plugs can be efficacious, especially with the recanalization subtype of persistence. The risk of persistence is thought to be minimized by deploying the coil as distally as possible. This becomes increasingly difficult as the anatomy becomes more tortuous and cannot be accessed with traditional 5- and 7-F coaxial catheters (5,25). Microcatheters become useful in this situation to help traverse difficult anatomy and obtain a more distal coil embolization (28). Despite this, the microcatheter group did not have lower rates of persistence.

Finally, this study demonstrated the low morbidity associated with PAVM embolization. In terms of the complications, paradoxical (ie, systemic) embolization occurred in one of 151 (0.7%) procedures. Some studies (29,30) reported that paradoxical embolization occurred in about 1%–2% of pulmonary AVM embolizations because of the high-flow nature of the PAVM. The use of detachable coils can help prevent complications caused by migrated coils.

Our study had limitations including its retrospective nature with its associated data limitations. Another limitation was the small sample size of the smoking group in this study. Verification of these results in a multicenter study would provide further validation of our findings. Finally, an additional limitation is that some patients may not have accurately described their smoking history or their smoking history was inaccurately documented in the medical record.

Patients with hereditary hemorrhagic telangiectasia (HHT) and active tobacco use have higher rates of pulmonary arteriovenous malformation (PAVM) persistence after percutaneous embolization. Smoking is a potentially modifiable risk factor for PAVM embolization persistence in patients with HHT after embolotherapy. These findings, if confirmed in larger studies, raise the possibility that smoking has an important role in persistence in patients with HHT.

## APPENDIX

Appendix E1 (PDF)
