# Supplementary material for: Smoking Significantly Impacts Persistence Rates in Embolized Pulmonary Arteriovenous Malformations in Patients with Hereditary Hemorrhagic Telangiectasia
Source: Radiology. 2019 Jul 30;292(3):762–70. doi: 10.1148/radiol.2019180978 (PMC6735354; doi:10.1148/radiol.2019180978)
Supplement: Appendix E1 (PDF) [file ry180978suppa1.pdf]

©RSNA, 2019
10.1148/radiol.2019180978

Appendix E1

CT Scanning Protocol

Imaging was performed on at least a Siemens Somatom Sensation 64-slice Scanner (Siemens, Munich, Germany). Images were performed in a single breath hold with the patient supine and arms above the head. Image acquisition parameters included a 0.5 pitch, 120 kVp, 250 mAs, and 24 × 1.2 mm collimation. Scans were triggered when the main pulmonary artery reached an attenuation of 100 Hounsfield units. Scan coverage starts from the top of lungs and extends to the bottom of the lungs. Images were reconstructed at 1.5 mm axial, 3.0 mm coronal, 3.0 mm sagittal, and 10.0 mm maximum intensity projection.
